# Is a perceived supportive physical environment important for self-reported leisure time physical activity among socioeconomically disadvantaged women with poor psychosocial characteristics? An observational study

**DOI:** 10.1186/1471-2458-13-280

**Published:** 2013-03-27

**Authors:** Verity J Cleland, Kylie Ball, David Crawford

**Affiliations:** 1 Centre for Physical Activity & Nutrition Research, Deakin University, 221 Burwood Hwy, Burwood, Victoria 3125, Australia; 2 Current address: Menzies Research Institute Tasmania, Private Bag 23, Hobart, Tasmania, 7001, Australia

**Keywords:** Social-ecological model, Health behaviour, Socioeconomic disadvantage, Cross-sectional, Survey research

## Abstract

**Background:**

Over the past decade, studies and public health interventions that target the physical environment as an avenue for promoting physical activity have increased in number. While it appears that a supportive physical environment has a role to play in promoting physical activity, social-ecological models emphasise the importance of considering other multiple levels of influence on behaviour, including individual (e.g. self-efficacy, intentions, enjoyment) and social (e.g. social support, access to childcare) factors (psychosocial factors). However, not everyone has these physical activity-promoting psychosocial characteristics; it remains unclear what contribution the environment makes to physical activity among these groups. This study aimed to examine the association between the perceived physical environment and self-reported leisure-time physical activity (LTPA) among women living in socioeconomically disadvantaged areas demonstrating different psychosocial characteristics.

**Methods:**

In 2007–8, 3765 women (18–45 years) randomly selected from low socioeconomic areas in Victoria, Australia, self-reported LTPA, and individual, social and physical environmental factors hypothesised within a social-ecological framework to influence LTPA. Psychosocial and environment scores were created. Associations between environment scores and categories of LTPA (overall and stratified by thirds of perceived environment scores) were examined using generalised ordered logistic regression.

**Results:**

Women with medium and high perceived environment scores had 20-38% and 44-70% greater odds respectively of achieving higher levels of LTPA than women with low environment scores. When stratified by thirds of psychosocial factor scores, these associations were largely attenuated and mostly became non-significant. However, women with the lowest psychosocial scores but medium or high environment scores had 76% and 58% higher odds respectively of achieving ≥120 minutes/week (vs. <120 minutes/week) LTPA.

**Conclusions:**

Acknowledging the cross-sectional study design, the findings suggest that a physical environment perceived to be supportive of physical activity might help women with less favourable psychosocial characteristics achieve moderate amounts of LTPA (i.e. ≥120 minutes/week). This study provides further support for research and public health interventions to target perceptions of the physical environment as a key component of strategies to promote physical activity.

## Background

Women of childbearing age and those experiencing socioeconomic disadvantage are population groups at high risk for physical inactivity [1,2] and consequently poor health and chronic disease. In particular, women experiencing socioeconomic disadvantage are at particular risk of inadequate leisure-time physical activity, with physical activity likely to be undertaken as a matter of necessity rather than choice [3]. As such, women experiencing socioeconomic disadvantage are important targets for physical activity promotion. In order to effectively target policies and programs, an understanding of the influences on physical activity is necessary. Social-ecological models (e.g. [4,5]) provide useful frameworks for understanding drivers of health behaviours such as physical activity. These models acknowledge multiple layers of influence, and propose that a range of individual, social and physical environment factors interact to influence behaviour [4,5]. Typically Social Ecological models do not specifically characterize the relationship of the environment with the individual, and hence investigations of different models incorporating both psychosocial and environmental factors simultaneously can help further elucidate associations among factors predicting behaviours such as physical activity, and advance social ecological theories of behaviour.

Many individual and social factors (termed here ‘psychosocial factors’, ie encompassing both psychological and social influences) have been identified as important predictors of physical activity behaviour (e.g. self-efficacy, enjoyment, intentions, skills, childcare, social support, dog ownership [6-8]), as have environmental factors (e.g. facilities, destinations, walkability, safety, sidewalks [9]). However, many women, particularly those experiencing socioeconomic disadvantage, lack ‘physical activity-promoting’ individual and social characteristics. For example, a number of women do not have high levels of self-efficacy, may not enjoy physical activity, or may not have the skills to be active; they may have unsupportive family and friends, or have no access to childcare [6,7]. It is important to recognise that many women do not possess these factors, because achieving improvements in these characteristics that result in increases in physical activity can be difficult [10,11]. The contribution of the physical environment to physical activity amongst these women and how this compares to those with more favourable psychosocial characteristics is unknown. A better understanding of how these factors relate to physical activity may provide insights for public health intervention – that is, are there things we can do at the environmental level that may impact on physical activity behaviour, irrespective of individual- and social-level factors?

While we and others’ have previously demonstrated that there are associations between individual, social and environmental factors and physical activity separately [8,12,13], no previous research has examined whether there are differential effects on physical activity when there are ‘mismatches’ between psychosocial- and environmental-level characteristics. The study aimed to examine the patterns of associations between perceptions of the physical environment and leisure-time physical activity (LTPA) among socioeconomically disadvantaged women stratified by different levels of psychosocial variables.

## Methods

Data were collected in the Resilience for Eating and Activity Despite Inequality (READI) study during 2007–8 [14,15]. The Deakin University Human Research Ethics Committee approved this study, with written informed consent obtained.

### Participants

Postcodes in the bottom third of the Socio-Economic Index for Areas (SEIFA) [16] distribution in Victoria, Australia, were classified as ‘disadvantaged’. Neighbourhoods scoring in the bottom third of this aggregate index are characterized, for example, by higher proportions of residents with relatively low incomes, low education levels, and higher rates of unemployment. Forty urban and 40 rural areas were randomly selected. From the Australian electoral roll (compulsory from age 18), 150 women (18–45 years) were randomly identified in each area (n = 11,940; some areas had <150 eligible women). After excluding 861 ‘return to sender’ surveys, 4,938 (45%) women completed a postal questionnaire. Excluded were those moving from the sampled suburb prior to survey completion (n = 571), surveys completed by a non-intended person (n = 3), those withdrawing data (n = 2), or those <17- or >46-years old (n = 13). Of the remaining participants (n = 4,349), those pregnant (n = 210); with missing data for pregnancy (n = 40), physical activity (n = 165), or environment (n = 97); or with >1 missing psychosocial factor (n = 22) or demographic factor (n = 74) were excluded, leaving 3,765 participants.

### Measures

#### Leisure time physical activity

Participants reported past week physical activity duration, frequency and intensity within leisure, transportation, occupational and domestic domains in the International Physical Activity Questionnaire (long version), which has demonstrated high reliability and acceptable validity across at least 12 countries [17]. Leisure time physical activity (LTPA) is discretionary and under volitional control (unlike for example, occupational activity) and therefore more likely to be amenable to intervention. In addition, LTPA conceptually is most likely to be influenced by local neighborhood environmental features (i.e., we would not expect to observe an association between perceived neighbourhood aesthetics and domestic activity, or between the local physical activity environment and occupational activity) [18]. For these reasons, LTPA was chosen as the focus for this paper, and included time spent walking for leisure, as well as moderate- and vigorous-intensity physical activity for leisure. Minutes/week of LTPA were calculated, then categorised because of high skewness resulting from the large number of zero values (a suitable transformation could not be determined). Domain-specific physical activity guidelines currently do not exist, so a categorical variable was calculated by classifying LTPA as 0 minutes/week (to account for the large number of zero values), then using tertile cutpoints to categorise the non-zero data as 1–119 minutes/week, 120–279 minutes/week, and ≥280 minutes/week. Tertiles were selected as they allow for an acceptable number of participants in each category while retaining sensitivity.

#### Psychosocial score

A ‘psychosocial score’ was created using four individual (self-efficacy, enjoyment, intentions, behavioural skills) and four social (child care, family support, friend support, dog ownership) variables previously found to be associated with LTPA in this [8] and other [2,13,19] samples. Where scales were used, internal reliability as assessed in this sample is presented as Cronbach’s alpha. Participants rated their confidence (self-efficacy) on a five-point Likert scale in being active in five difficult circumstances [20]; responses were summed (Cronbach’s alpha = 0.82). Responses to six items on a seven-point Likert scale about physical activity enjoyment [21] were summed (Cronbach’s alpha = 0.95). A seven-point Likert scale was used to assess activity intentions [22], and behavioural skills were assessed by summing two questions about past week frequency of goal setting and planning for physical activity [22] (Cronbach’s alpha = 0.83). Participants reported access to childcare if they wanted to be active (not applicable, yes, no), and dog ownership (yes, no). Family social support was assessed by asking how often in the past year family members engaged in physical activity with them or encouraged physical activity [23]; responses to the two questions were summed (Cronbach’s alpha = 0.76). The same questions were asked in reference to friends/work colleagues (Cronbach’s alpha = 0.69).

Childcare was scored 0 (not available) or 1 (available or not applicable), and dog ownership was scored 0 (no dog) or 1 (dog owner). To overcome differences in scales, each of the other four individual and two social variables were categorised into thirds and allocated a score of 0 (lowest third), 1 (middle third) or 2 (highest third). Scores for the eight variables were summed to create an overall ‘psychosocial score’, with a possible range of 0–14. This score was classified as low (0–6), medium (7–9) or high (10–13) based on tertile cutpoints, with higher scores indicating more favourable psychosocial characteristics. While some information on specific variables may be lost, this approach provides an opportunity to categorise discordant participant psychosocial ‘profiles’ in a parsimonious manner. That is, a range of psychosocial variables are incorporated into a single score, providing an aggregate indicator of psychosocial support, and reducing complexity and issues associated with multiple testing of numerous constructs in isolation. The internal consistency of the psychosocial score was reasonable (Cronbach’s alpha = 0.66).

#### Perceived environment score

A perceived neighbourhood ‘environment score’ was created from three self-reported variables described in our earlier work [8]. Briefly, personal safety (sum of three items; Cronbach’s alpha = 0.85), aesthetics (sum of five items; Cronbach’s alpha = 0.76), and the ‘physical activity environment’ (sum of seven items; Cronbach’s alpha = 0.80) were assessed on five-point Likert scales adapted from an existing measure [24]. To overcome differences in scales, each of the three environmental variables was categorised into thirds and allocated a score of 0 (lowest third), 1 (middle third) or 2 (highest third). Scores for the three variables were summed to create the score, with a possible and actual range of 0–6. This score was classified as low (0–1), medium (2–3) or high (4–6) based on tertile cutpoints, with higher scores indicating more favourable physical environments. The internal consistency of the environment score was reasonable (Cronbach’s alpha = 0.63).

#### Potential covariates

Self-reported demographic characteristics included age; marital status (married/living as married, previously married, or not married); level of education (low = less than Year 12; mid = Year 12, certificate/trade/diploma; high = tertiary); employment status (full-time work, part-time work, or not employed); children <18 years (none, 1, 2, 3+); birth country (Australia or outside Australia); height and weight, converted to body mass index (BMI) and categorised as healthy (including underweight), overweight or obese (≤25 kg/m^2^, 25–29.9 kg/m^2^, ≥30 kg/m^2^, respectively) [25]; presence of illness/injury/disability likely to affect physical activity (yes, no); current smoking (yes, no); and access to a motor vehicle (yes, no). Area of residence (urban/rural) was assessed as a potential covariate. Urban and rural are defined elsewhere in detail (Cleland, Hume, et al., 2010). Briefly, urban areas included metropolitan Melbourne, rural cities (defined by the Regional Infrastructure Development Act 1999 [Vic]) and all suburbs completely within a 10 km radius of the centroid of these cities, and all suburbs completely within a 10 km radius of the centroid of other Victorian cities with a population of ≥20,000. Rural areas were those falling outside of metropolitan Melbourne and outside of a 25 km radius of the rural cities.

#### Analyses

Demographic characteristics (potential covariates) are presented for the overall sample and across categories of LTPA. Differences across these categories were tested using one-way analysis-of-variance (equal variances) or Kruskal-Wallis equality-of-populations rank test (unequal variances) for continuous variables, and Pearson’s chi-square test for categorical variables. Mean (and standard deviation) and median (and inter-quartile range) LTPA is presented overall and across categories of environment score categories (low, medium, high) and psychosocial score categories (low, medium, high). Cuzick’s non-parametric test for trend was used to assess whether a significant trend was evident for LTPA across increasing environment score categories and across increasing psychosocial score categories.

Because of the ordered but not evenly spaced nature of the outcome data, generalised ordered logistic regression was employed by using the ‘gologit2’ command [26] in Stata, Version 10.2 (Statacorp, College Station, Texas) to calculate odds ratios (OR) and 95% confidence intervals (CI) for increasing categories of LTPA across categories of the environment score. These analyses were repeated stratified by psychosocial score categories to examine the association between the environment and LTPA across categories of psychosocial scores. In generalised ordered logistic regression, the actual values of the dependent variable (LTPA) are irrelevant except that it is assumed that larger values correspond to ‘higher’ outcomes. The results indicate the odds of participating in increasing LTPA associated with one-unit increases in the independent variables (e.g. the difference between a low and medium environment score). Unlike standard logistic regression where increasing categories are compared to a typical ‘referent’ group (e.g. the bottom/lowest category), ordered logistic regression compares each increasing outcome category to all data falling below the cutpoint for that category (e.g. <120 minutes/week is the referent group for calculating the odds of achieving ≥120 minutes/week LTPA, while <280 minutes/week is the referent group for calculating the odds of achieving ≥280 minutes/week LTPA). Demographic characteristics that were associated with LTPA and the environment score were included as confounders in regression models. Analyses were conducted in 2010 and included adjustment of the standard errors for the effects of clustering within the 80 areas (the unit of recruitment).

## Results

More active women were younger, had lower BMI values, had higher levels of education, were employed full-time, were not married, had no children, were Australian-born, were non-smokers, did not have an illness/injury/disability preventing physical activity, and were a healthy weight (Table 1). Overall, women reported a median of 2 hours per week LTPA (Table 2). LTPA was significantly higher across increasing environment score categories and across increasing psychosocial score categories.

**Table 1:** Socio-demographic and health characteristics of women in the READI study by LTPA

	**Overall**	**LTPA**			
		**0 mins/week (n = 1021)**	**1-119 mins/wk (n = 762)**	**120-279 mins/wk (n = 1034)**	**≥280 mins/wk (n = 948)**
Age (years), *M (SD)*	34.6 (8.2)	35.7 (7.9)	34.0 (8.1)	34.4 (8.4)	34.1 (8.4)**
BMI (kg/m^2^), *M (SD)*	26.0 (6.1)	27.2 (7.0)	25.8 (5.9)	25.7 (5.5)	25.3 (5.6)**
Level of education, *n (%)*					
Low	833 (22.3)	317 (31.3)	139 (18.3)	206 (20.0)	171 (18.2)**
Medium	1937 (51.7)	520 (51.3)	404 (53.3)	510 (49.5)	503 (53.4)
High	974 (26.0)	176 (17.4)	215 (28.4)	315 (30.6)	268 (28.5)
Employment status, *n (%)*					
Working full-time	1426 (38.6)	361 (36.1)	272 (36.4)	392 (38.4)	401 (43.4)**
Working part-time	1091 (29.5)	272 (27.2)	227 (30.4)	309 (30.2)	283 (30.6)
Not employed	1179 (31.9)	368 (36.8)	249 (33.3)	321 (31.4)	241 (26.1)
Access to a motor vehicle, *n (%)*	3,517 (93.7)	948 (93.0)	713 (93.6)	959 (93.3)	897 (94.9)
Marital status, *n (%)*					
Not married	1000 (26.6)	247 (24.2)	184 (24.2)	277 (26.8)	292 (30.9)*
Married/Living as married	2443 (65.0)	667 (65.4)	528 (69.3)	671 (65.0)	577 (61.0)
Previously married	317 (8.4)	106 (10.4)	50 (6.6)	84 (8.1)	77 (8.1)
No. of children <18 yrs, *n (%)*					
None	1464 (39.3)	328 (32.6)	287 (38.2)	410 (40.0)	439 (46.8)**
One	661 (17.8)	215 (21.4)	116 (15.4)	188 (18.3)	142 (15.1)
Two	968 (26.0)	280 (27.8)	208 (27.7)	261 (25.5)	219 (23.3)
≥Three	629 (16.9)	183 (18.2)	141 (18.8)	166 (16.2)	139 (14.8)
Urban area of residence, *n (%)*	1735 (46.1)	496 (48.6)	341 (44.8)	471 (45.6)	427 (45.0)
Born in Australia, *n (%)*	3371 (89.6)	883 (86.5)	687 (90.3)	933 (90.2)	868 (91.6)*
Current smoker, *n (%)*	608 (16.2)	269 (26.4)	110 (14.5)	124 (12.0)	105 (11.1)**
Illness/injury/disability, *n (%)*	425 (11.3)	152 (14.9)	91 (12.4)	87 (8.4)	91 (9.7)**
Weight status, *n (%)*					
Healthy weight	1913 (53.1)	442 (45.8)	401 (54.4)	536 (54.0)	534 (58.8)**
Overweight	912 (25.3)	238 (24.7)	189 (25.6)	271 (27.3)	214 (23.5)
Obese	778 (21.6)	285 (29.5)	147 (20.0)	185 (18.7)	161 (17.7)

*READI* resilience for eating and inactivity despite inequality study; *SD* standard deviation; *BMI* body mass index; *LTPA* leisure time physical activity.

**p < 0.001, *p < 0.01 for differences across categories of LTPA from one-way analysis-of-variance or Kruskal-Wallis equality-of-populations rank test (continuous variables) or Pearson’s chi-square test (categorical variables).

**Table 2:** LTPA (minutes/week) overall and stratified by environment score and psychosocial score*, among women in the READI study

**Leisure time physical activity**	**n (%)**	**Mean (SD)**	**Median (IQR)**
Overall	3765 (100%)	206.3 (272.9)	120 (0, 280)
Environment score			
Low	1064 (28.3)	181.2 (269.7)	90 (0, 240)
Medium	1159 (30.8)	204.7 (270.5)	120 (0, 270)
High	1542 (41.0)	224.9 (275.5)	150 (30, 300)
*p*_*trend*_		**<.001**	
Psychosocial score			
Low	1126 (29.9)	81.8 (175.1)	0 (0, 90)
Medium	1131 (30.0)	174.7 (238.7)	90 (0, 240)
High	1508 (40.1)	323.0 (307.2)	240 (120, 420)
*p*_*trend*_		**<.001**	

*SD* standard deviation; *IQR* inter-quartile range; *READI* resilience for eating and inactivity despite inequality study.

*p-values from Cuzick non-parametric test for trend comparing leisure time physical activity values across increasing categories.

Higher environment factor scores were associated with increasing odds of higher levels of LTPA (Table 3). Compared with those with low environment scores, those with medium and high environment scores had 38% and 67% greater odds respectively of doing any LTPA (compared with no LTPA), 37% and 70% greater odds respectively of ≥120 minutes/week LTPA (compared with <120 minutes/week LTPA), and 20% and 44% higher odds respectively of ≥280 minutes/week LTPA (compared with <280 minutes/week LTPA).

**Table 3:** **ORs**^**a **^**for increasing LTPA by environment score and psychosocial score, among women in the READI study**

	**LTPA**		
	**≥1 min/wk**	**≥120 mins/wk**	**≥280 mins/wk**
	**(vs. 0 min/wk)**	**(vs. <120 mins/wk)**	**(vs. <280 mins/wk)**
*Environment score*			
Low (n = 1009)	1.0 (reference)	1.0 (reference)	1.0 (reference)
Med (n = 1112)	1.38 (1.15 1.64)**	1.37 (1.18, 1.59)**	1.20 (1.01, 1.43)*
High (n = 1495)	1.67 (1.40, 2.00)**	1.70 (1.46, 1.98)**	1.44 (1.22, 1.70)**
*p*_*trend*_	<0.001	<0.001	<0.001
*Psychosocial score*			
Low (n = 1075)	1.0 (reference)	1.0 (reference)	1.0 (reference)
Medium (n = 1088)	3.52 (2.89, 4.30)***	2.81 (2.36, 3.34)***	2.72 (2.05, 3.61)***
High (n = 1453)	15.31 (12.42, 18.88)***	11.32 (9.55, 13.42)***	8.20 (6.42, 10.49)***
*p*_*trend*_	<0.001	<0.001	<0.001

*LTPA* leisure time physical activity, *READI* resilience for eating and inactivity despite inequality study; * p < 0.05 ** p < 0.01 *** p < 0.001.

^a^ Odds ratios and 95% confidence intervals from generalised ordered logistic regression model adjusted for age, number of children, country of birth, employment status, marital status, smoking status, and injury/illness/disability; robust standard errors adjusting for clustering by neighbourhood (the unit of recruitment). Generalised ordered logistic regression is appropriate for the ordered but not evenly spaced outcome data, and compares each increasing category to all data below the cut-point for that category.

When stratified by psychosocial score category, the association between the environment score and LTPA was largely attenuated except among women with low psychosocial scores (Table 4). Among these women, compared with those with low environment scores, women with medium or high environment scores had 76% (medium environment score) and 58% (high environment score) greater odds of ≥120 minutes/week of LTPA (compared with <120 minutes/week LTPA).

**Table 4:** **OR**^**a **^**for LTPA according to environment score, stratified by psychosocial score, among women in the READI study**

**Environment score by psychosocial score**	**LTPA**		
	**≥1 min/wk (vs. 0 min/wk)**	**≥120 mins/wk (vs. <120 mins/wk)**	**≥280 mins/wk (vs. <280 mins/wk)**
	**OR (95% CI)**	**OR (95% CI)**	**OR (95% CI)**
*Low psychosocial score*			
Low environment score (n = 402)	1.0 (reference)	1.0 (reference)	1.0 (reference)
Med environment score (n = 350)	1.21 (0.88, 1.65)	1.76 (1.22, 2.54)*	1.34 (0.75, 2.37)
High environment score (n = 323)	1.19 (0.87, 1.65)	1.58 (1.07, 2.34)*	1.38 (0.80, 2.40)
*Med psychosocial score*			
Low environment score (n = 316)	1.0 (reference)	1.0 (reference)	1.0 (reference)
Med environment score (n = 342)	1.14 (0.80, 1.64)	0.92 (0.70, 1.22)	0.78 (0.54, 1.14)
High environment score (n = 430)	0.94 (0.66, 1.32)	0.91 (0.67, 1.23)	0.79 (0.54, 1.16)
*High psychosocial score*			
Low environment score (n = 291)	1.0 (reference)	1.0 (reference)	1.0 (reference)
Med environment score (n = 420)	1.29 (0.76, 2.20)	1.08 (0.76, 1.54)	1.11 (0.80, 1.54)
High environment score (n = 742)	1.24 (0.73, 2.10)	1.14 (0.81, 1.60)	1.10 (0.81, 1.47)

*LTPA* leisure time physical activity, *READI* resilience for eating and inactivity despite inequality; * p < 0.05.

^a^ Odds ratios and 95% confidence intervals from generalised ordered logistic regression model adjusted for age, number of children, country of birth, employment status, marital status, smoking status, and injury/illness/disability; robust standard errors adjusting for clustering by neighbourhood (the unit of recruitment). Generalised ordered logistic regression is appropriate for the ordered but not evenly spaced outcome data, and compares each increasing category to all data below the cut-point for that category.

## Discussion

This study examined associations between perceptions of the physical environment and LTPA among socioeconomically disadvantaged women possessing varying levels of individual and social factors known to be associated with physical activity. The findings suggest that a physical environment perceived to be supportive was associated with moderate levels of LTPA amongst women with psychosocial characteristics considered less beneficial for physical activity. This relationship was not evident among women with more favourable psychosocial characteristics, suggesting that perceiving a supportive physical environment may be particularly important for women with less favourable psychosocial characteristics.

Among women with low psychosocial scores, the perceived physical environment was associated with moderate amounts of LTPA (120 minutes/week) but not with any (versus none) or high LTPA (≥280 minutes/week). This could be due to the categorisation of LTPA and the individual, social and environmental factors, which may have reduced precision to detect associations, although descriptive analyses identified associations in the expected directions. Alternately, these women may undertake activities that can be performed in their local neighbourhood (such as walking), and as a result are more aware of the characteristics of their physical environment. More active women may perform different types of physical activity (e.g. organised sport, or walking, running or cycling longer distances) undertaken outside the neighbourhood, reducing their awareness of local neighbourhood characteristics. This is plausible as for women reporting 120–279 minutes/week LTPA, a greater proportion comprised walking (62%) compared with women reporting ≥280 minutes/week (53%). Another Australian study also found a greater proportion of participants walked for recreation within their neighbourhood than outside the neighbourhood, and that recreational walking within the neighbourhood was of greater duration than recreational walking outside the neighbourhood [27]. This suggests that amongst women with low psychosocial scores, perceiving a supportive environment may be important for achieving levels of LTPA that approximate the recommended 150 minutes/week [28,29]. Plausibly, women who have high psychosocial scores may be active irrespective of their environment, whereas for women with lower psychosocial scores, perceiving a supportive environment may be necessary to influence LTPA.

This study is limited by its cross-sectional design. It is plausible that active women perceive their environments as more supportive of activity or are more aware of their physical environment, or that women with more favourable psychosocial characteristics (e.g. higher physical activity self-efficacy) may over-optimistically report physical activity. Self-reported measures of LTPA and environments may limit findings. However, there was variability within psychosocial and environmental score categories, and associations were observed with well-established correlates (e.g. age, BMI, education, employment status, number of children) [2] in expected directions. While a reliable and valid measure of physical activity was used (the IPAQ-L) [17], the psychometric properties of specific domains of activity such as during leisure time, have not been assessed. Lack of specificity in the environmental measures in relation to LTPA might have reduced the ability to detect associations [18]. For instance, the neighbourhood ‘physical activity environment’ refers mostly to walking activities, but the measure of LTPA is not limited to walking. However, walking is the most common leisure activity amongst Australian women [30] and more than 60% of participants reported walking for leisure. The large number of comparisons may be considered a limitation. However, by reducing the likelihood of false positives (a type I error) by adjusting for multiple comparisons, the likelihood of false negatives (a type II error) is increased, offering no real improvement [31].

While lack of access to a motor vehicle may have impacted the ability to access other more supportive environments further afield, only 6% of participants reported not having access to a motor vehicle (car ownership is high in Australia, with approximately 740 registered passenger vehicles per 1000 residents in Victoria [32]) access to a motor vehicle did not meet the definition of a confounder. We did not adjust for income, but previous analyses of data from this study found no association between income and LTPA [33], and hence income did not meet our definition of a confounder and was not considered as a potential covariate. It is plausible that there was residual confounding from the use of self-reported measures, unmeasured variables, or variables not included in our analyses.

This study was novel in its approach to understanding the complex relations between perceived individual, social and environmental factors and LTPA. While previous research has examined associations between individual, social and environmental factors and physical activity [8,12,13], no studies have examined the effect on physical activity when there are ‘mismatches’ in levels of individual, social and environmental characteristics. Other strengths include the large sample size which enabled assessment of a range of factors simultaneously while adjusting for known covariates and stratification by variables of interest, appropriate statistical adjustment for the multilevel data structure, and the use of a theoretical framework to guide selection of correlates. Our work focused on an under-studied population group at high risk for inactivity and chronic disease, and provides insights for targeting and development of interventions to promote LTPA among women living in socioeconomically disadvantaged areas.

## Conclusion

In conclusion, the findings suggest that for women with less favourable psychosocial characteristics (e.g. lower self-efficacy for physical activity, fewer intentions to be active, poorer social support networks), improving perceptions of the physical environment may be a worthwhile avenue for public health initiatives to increase physical activity. The findings also suggest that perceiving a supportive physical environment may make little difference to the likelihood of physical activity levels among women who already possess more favourable psychosocial characteristics (e.g. high self-efficacy for physical activity, greater intentions to be active, better social support networks). Strategies focused on improving general perceptions of the physical environment (e.g. promotion of facilities) may be warranted.

## Abbreviations

CI: Confidence interval; LTPA: Leisure-time physical activity; OR: Odds ratio; READI: Resilience for eating and inactivity despite inequality.

## Competing interests

The authors declare that they have no competing interests.

## Authors’ contributions

VC conceptualised the research question, conducted the analyses and drafted the manuscript. KB made substantial contributions to the conception and design of the study and research question, interpretation of data, and critical revision of the manuscript. DC made substantial contributions to the conception and design of the study, interpretation of data, and critical revision of the manuscript. All authors read and approved the final manuscript.

## Pre-publication history

The pre-publication history for this paper can be accessed here:

http://www.biomedcentral.com/1471-2458/13/280/prepub
